# Surgical treatment of acromioclavicular dislocation using the endobutton

**DOI:** 10.1590/1413-785220172503166657

**Published:** 2017

**Authors:** Renato Loureiro Teodoro, Alexandre Yukio Nishimi, Luciano Pascarelli, Roberto Rangel Bongiovanni, Marcelo Andreotti Perez Velasco, Eiffel Tsuyoshi Dobashi

**Affiliations:** 1 Hospital IFOR - Rede D´Or, Shoulder and Elbow Surgery Group, São Bernardo do Campo, SP, Brazil; 2 Hospital IFOR - Rede D´Or, Pediatric Orthopedics, São Bernardo do Campo, SP, Brazil

**Keywords:** Acromioclavicular joint/diagnostic imaging, Acromioclavicular joint/surgery, Ligaments articular, Orthopedic procedures/methods, Evaluation studies

## Abstract

**OBJECTIVE:**

To evaluate the clinical and radiographic results of 23 patients diagnosed with acute type III acromioclavicular dislocation treated with the Endobutton.

**METHODS:**

Twenty-three patients with a diagnosis of type III acromioclavicular dislocation were treated surgically.

**RESULTS:**

Twenty-one patients were male (91.3%) and 2 (8.7%) were female. The dominant side was affected in 15 patients (65.21%) and the non-dominant side in 8 patients (34.79%). All patients were operated on by the same surgical team within 4 weeks of the trauma. According to the UCLA score, 14 patients (60.86%) presented excellent results, 7 patients (30.43%) had good results and 2 patients (8.69%) had regular results.

**CONCLUSION:**

The technique was effective in treating acute type III dislocations with a high degree of patient satisfaction.***Level of Evidence IV, Case Series.***

## RESUMO

**OBJETIVO:**

Avaliar os resultados clínicos e radiográficos de 23 pacientes com diagnóstico de luxação acromioclavicular aguda tipo III tratados com uso de placa Endobutton.

**MÉTODOS:**

Foram submetidos a tratamento cirúrgico 23 pacientes com diagnóstico LAC III.

**RESULTADOS:**

O sexo masculino foi prevalente, sendo 21 (91,3%) homens e duas (8,7%) mulheres. O lado dominante foi acometido em 15 pacientes (65,21%) e o não dominante, em oito pacientes (34,79%). Todos os pacientes foram operados pela mesma equipe cirúrgica em até quatro semanas da data do trauma. Pelo escore da UCLA: 14 pacientes (60,86%) apresentaram excelentes resultados, sete pacientes (30,43%), bons resultados e em dois pacientes (8,69%) os resultados foram regulares.

**CONCLUSÃO:**

A técnica mostrou-se efetiva no tratamento das luxações agudas de grau III, com elevado grau de satisfação dos pacientes. ***Nível de Evidência IV, Série de Casos.***

## INTRODUCTION

Dislocation of the acromioclavicular joint (ACJ) is a common injury^1^ responsible for 9% of all shoulder injuries,^2^ and is one of the oldest traumatic pathologies recorded in the literature. It is 10 times more common in males^3^ from 20 to 39 years of age^4^ and predominant in young people since it is associated with high-impact sports and high-speed vehicle accidents.^5^ ^,^ ^6^

The Rockwood classification^7^ is most commonly used to describe the degree of injury, which was initially described by Tossy et al.,^8^ who divided acromioclavicular dislocations into three types: types I and II are light to moderate and are treated conservatively, while type III is severe and involves an offset of at least 1 cm or larger than the thickness of the clavicle, requiring surgical treatment. The Rockwood classification^7^ modified type III and added the types IV, V and VI to Tossy's classification.

After a traumatic episode, diagnosis is made clinically and using x-rays. The clinical examination is characterized by the presence of sharp pain in the upper portion of the shoulder, algic limitation, edema, bruising, deformity and piano key sign (clavicle is reduced by pressing downward on the deformed area like pushing down a piano key, a sign present in most type III and V ACJ dislocations). Characteristic radiographic findings seen in the AP view (or in the Zanca view for better definition of the ACJ) permit the vertical stability of the ACJ to be assessed and the axillary view permits assessment of the horizontal stability of the ACJ.

ACJ dislocation has been a subject of controversy since the time of Hippocrates (460-377 BC). Treatment of Rockwood types I and II initially do not involve surgery;^9^ treatment of type III remains controversial and may be approached using the criteria by Copeland:^10^

- Conditions in favor of: young, slim, athletic patient, manual laborer, dominant side, joint cannot be reduced.
- Conditions against surgery: elderly, obese, sedentary patient, non-manual laborer, non-dominant side, reducible joint and little apparent deformity.

Types IV, V and VI require surgical treatment.^11^ The techniques may involve the use of wires, transfer of the coracoacromial ligament, fixing the coracoacromial interval with a screw, suture anchors, or suture loops, reconstruction of the acromioclavicular and coracoclavicular ligaments and using the Endobutton and may be conducted in open or minimally invasive procedures or use arthroscopy.

The open approach is most common; its advantages include direct visualization of the ACJ, the possibility to remove any degenerative material from the disk, precise identification of the origins of the coracoclavicular ligaments and shorter surgical time. Disadvantages include a more prominent scar and aggression to the insertion of the deltoid muscle.^12^ ^,^ ^13^

The objective of this study was to evaluate the clinical and radiographic results of 23 patients diagnosed with acute Rockwell type III ACJ dislocation who were treated with coracoclavicular fixation using two anchors and an Endobutton using the open approach.

## MATERIALS AND METHODS

This study was approved by the institutional review board under process number 1717404. All patients participating in the study signed the terms of free and informed consent. From June 2013 to March 2015 23 patients underwent surgical treatment for ACJ dislocation. Mean patient age was 31.04 years (minimum 19 years; maximum 45 years). Males were more prevalent, with 21 patients (91.3%) compared with 2 (8.7%) females. The dominant side was affected in 15 patients (65.21%) and the non-dominant side in 8 patients (34.79%). All patients were operated by the same surgical team until 4 weeks of the date of the trauma. Minimum follow-up was 6 months.

**Figure 1:**
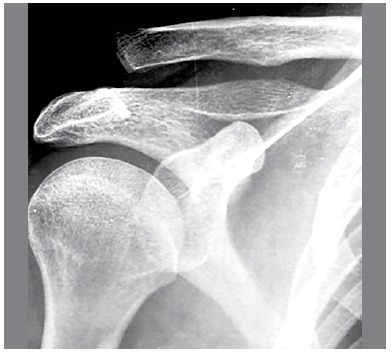
X-*ray*, AP view of right shoulder.

The study included patients with clinical and radiographic diagnosis of Rockwood type III ACJ dislocation with evolution of up to 4 weeks (average of 8 days). (Figure 1) Exclusion criteria were patients with osteoarthritis in the ACJ and injuries which occurred more than 4 weeks prior.

### Rockwood Classification:

Type I: sprain of the AC ligaments (35% of cases) - x-ray shows no alterations.

Type II: rupture of the acromioclavicular ligaments and sprain of the coracoclavicular ligaments (22% of cases) - x-ray shows <25% increase in acromioclavicular space.

Type III: rupture of the acromioclavicular and coracoclavicular ligaments (39% of cases) - x-ray shows 25-100% increase in acromioclavicular space.

Type IV: rupture of acromioclavicular ligaments - x-ray may show normal coracoclavicular space with posterior dislocation of the clavicle.

Type V: rupture of the acromioclavicular and coracoclavicular ligaments, desinsertion of the trapezoid and deltoid muscles in the distal half of the clavicle - x-ray shows a 100-300% increase in acromioclavicular space.

Type VI: rupture of acromioclavicular ligaments with inferior dislocation of the clavicle - x-ray shows inferior dislocation of the clavicle.

Tables 1, ^2^ and ^3^ present the distribution of the operated patients according to age, sex and affected side, respectively.

**Table 1:** Patient age, highlighting youngest, oldest and mean.

Patient age		
Age	Years
Youngest	19
Oldest	45
Mean	31.04

**Table 2:** Patient distribution according to sex.

Sex distribution		
Limb	Frequency	%
Male	21	91.3
Female	2	8.7

**Table 3:** Patient distribution according to side affected.

Distribution, side affected			
Limb	Frequency	%
Dominant	15	65.21
Non-dominant	8	34.79

### UCLA Criteria for clinical evaluation. (Chart 1)

**Figure 2. X-ray,:**
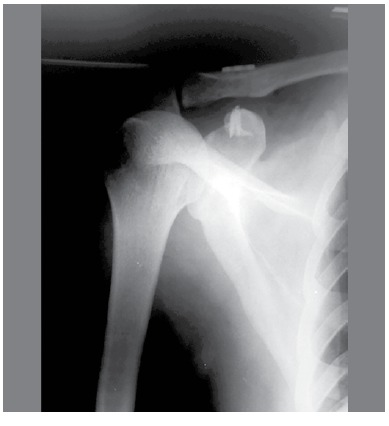
post-procedure.

Surgical technique: The patient is placed in "beach chair" position under general anesthesia and brachial plexus block is applied. Access occurs through an incision (about 3 cm) made topographically along the anterior edge of the clavicle (5 mm medial to the acromioclavicular joint) to the top edge of the coracoid process. The deltotrapezoidal fascia is opened and the deltoid detached to visualize the acromioclavicular joint and coracoid process. Two anchors are fixed at the base of the coracoid process. A downward hole is made in the clavicle using a 2.5 mm drill 3 cm medial to the acromioclavicular joint and equidistant between the anterior and posterior edges of the clavicle. The anchor wires are individually passed through the hole in the clavicle from bottom to top, using No. 1 Aciflex wire. After reduction of the ACJ, the Endobutton (composed of 4 holes) is placed on the hole and the lateral anchor wires are passed through the 1^st^ and 3^rd^ holes (hole order increases from lateral to medial), while the more medial anchor wires are passed through the 2nd and 4^th^ holes The tie-off is made separately from the anchor wires and reinsertion of the deltotrapezoidal muscles uses the anchor wires which are already attached. Intraoperative arthroscopy is conducted to visualize the reduction and x-rays are taken after the procedure. (Figure 2)

Patients are immobilized using a Velpeau sling for 4 weeks with immediate release of active flexion-extension of the elbow, wrist and hand.

Radiographic evaluation includes the anteroposterior, axillary and shoulder profile views to assess the comparative coracoclavicular distance and is conducted weekly during the first month and monthly until the sixth month.

**Chart 1:** Scoring results according to UCLA.

Pain	
Present all the time, unbearable, frequent use of strong painkillers	1
Present all the time, unbearable, occasional use of strong painkillers	2
Weak/absent at rest, present during light activity, frequent use of salicylates	4
Present during heavy/specific activities, frequent use of salicylates	6
Occasional and weak	8
Absent	10
Function	
Unable to use the limb	1
Only light activities possible	2
Able/activities at home/activities of daily living	4
Activities at home/shopping/driving/combing hair/dressing/put on clothing that closes in the back	6
Mild restriction/able to work above shoulder level	8
Normal activities	10
Active flexion	
150 degrees or more	5
120 to 150 degrees	4
90 to 120 degrees	3
45 to 90 degrees	2
30 to 45 degrees	1
Less than 30 degrees	0
Anterior flexion strength (manual muscle test)	
Grade 5 (normal)	5
Grade 4 (good)	4
Grade 3 (average)	3
Grade 2 (weak)	2
Grade 1 (muscle contractions)	1
Grade 0 (absent)	0
Patient satisfaction	
Satisfied and better	5
Unsatisfied and worse	0
Maximum score: 35 points	
Ellmann Score (UCLA)	
34-35	Excellent
28-33	Good
21-27	Reasonable
00-20	Poor

## RESULTS

According to UCLA score and Elmann criteria: 14 patients (60.86%) presented excellent results, 7 patients (30.43%) had good results and in 2 patients (8.69%) results were reasonable. All patients reported satisfaction with the treatment.

The patients were evaluated after 6 months. Five patients (21.73%) experienced less than 30% loss of reduction, but without functional impairment. In one patient (4.3%) there was superficial infection of the surgical wound, with resolution in 7 days.

## DISCUSSION

The ideal method for treating type III ACJ dislocation remains controversial in the literature. There are several surgical techniques and preferences described for treating the acromioclavicular joint.^14^

Fixation with Kirschner wires is not used often at present because of high rates of complications such as breakage and material migration, infection, arthritis and loss of reduction.^15^

The coracoacromial ligament transfer described in 1972 by Weaver and Dunn consists of deinsertion of the coracoacromial ligament from the acromium and transposing it into the intramedullary region of the distal portion of the clavicle. However, a complication of this technique is loss of joint reduction.^16^

The Bosworth technique uses a screw to affix the clavicle to the coracoid process. Although it is effective and restores the reduction of the ACJ, the screw may break or loosen or the coracoid process may fracture, requiring a new procedure to remove the material and presenting high rates of osteolysis in the clavicle.^17^ The subcoracoid ties with high-strength wires can cause bone erosion and anterior subluxation of the clavicle.^18^

Some authors performed arthroscopic reconstruction of the coracoclavicular ligament. The use of autologous semitendinous graft or synthetic suture^19^ has the advantage of preserving the deltoid insertion and this method also permits treatment of associated injuries and uses a minimally invasive route.

The use of fixation anchors on the coracoid process reduces the risk of neurovascular injury and decreases surgical time in comparison with the subcoracoid knot and avoids displacement of this tie-off to the anterior portion of the coracoid.^20^

The technique of stabilization between the clavicle and the coracoid process using the Endobutton or anchors has been described by various authors^20^ with satisfactory results. Its advantages include the fact that it is not necessary to remove synthetic material and it has been shown to be effective in restoring and maintaining the reduction of the ACJ. Complications include the cut-out of the suture, foreign body reaction (observed mainly when polytetrafluoroethylene suture is used) and potential osteolysis in the clavicle.

In our study, the technique permitted a small open approach with relatively short surgical time. The use of anchors allowed fixation at the base of the coracoid process in the region where the conoid ligament is inserted. The Endobutton was effective because there is no need to remove it; to avoid the shear effect of the wires with consequent failure of the synthesis resulting from passing through a single bone tunnel, they are tied over the Endobutton so that the knot does not come into contact with the clavicle.

The degree of satisfaction (excellent and good) among patients who underwent the Endobutton procedure in our study was 91%.

## CONCLUSION

The technique proved to be effective in treating acute ACJ dislocations (Rockwood type III) with a high degree of patient satisfaction.
